# Intra-articular dexmedetomidine in knee arthroscopy: A systematic review and meta-analysis

**DOI:** 10.1038/s41598-018-22482-8

**Published:** 2018-03-06

**Authors:** Ke Peng, Wei-rong Chen, Xiao-wen Meng, Juan Zhang, Fu-hai Ji

**Affiliations:** Department of Anesthesiology, First Affiliated Hospital of Soochow University, Suzhou, China

## Abstract

The aim of this meta-analysis is to evaluate the analgesic effects of intra-articular dexmedetomidine (DEX) in arthroscopic knee surgery. A comprehensive literature search was conducted to identify randomized controlled trials (RCTs) comparing intra-articular DEX versus control for postoperative analgesia in knee arthroscopy. Trial sequential analysis (TSA) was applied to determine the reliability of the evidence. Twelve RCTs including 594 patients met the eligibility criteria. DEX treatment significantly improved postoperative pain outcomes, with weighted mean differences (95% confidence interval) between the DEX and control groups of −1.57 (−1.94 to −1.20, P < 0.00001) for pain scores at rest at postoperative 1 h, −8.54 mg (−11.96 to −5.13, P < 0.00001) for morphine-equivalents at postoperative 0–24 h, and 257.57 min (209.86 to 305.28, P < 0.00001) for time to first request for postoperative analgesia. TSA indicated there is sufficient evidence for these outcomes. Intra-articular DEX did not affect the incidence of postoperative nausea and vomiting, hypotension, bradycardia, or somnolence. This meta-analysis demonstrated that intra-articular administration of DEX improved pain outcomes in the early postoperative period after knee arthroscopy. Due to the limited number of trials and patients included in this meta-analysis, more evidence is required to confirm these findings.

## Introduction

Arthroscopic knee surgery is a common orthopedic surgical procedure^1^. Patients are managed on a day-case basis; therefore, adequate postoperative pain relief is critical to facilitate patient discharge and early rehabilitation. A simple method of analgesia with a prolonged duration of action and minimal adverse effects is required.

Systemic dexmedetomidine (DEX) produces sedative, analgesic, sympatholytic, and anesthetic-sparing effects^2^. Evidence suggests that intra-articular administration of local anesthetics, opioids, magnesium, clonidine, and DEX, alone or in combination, decreases postoperative pain without significant adverse effects^3–7^. A combination of a local anesthetic agent and DEX, applied at the end of surgery, which targets peripheral nociceptive receptors may be an ideal protocol for pain control after knee arthroscopy.

To the author’s knowledge, there are no published meta-analyses investigating the effects of intra-articular administration of DEX on postoperative pain after knee arthroscopic procedures. This study was designed to determine the benefits and adverse effects of intra-articular administration of DEX in arthroscopic knee surgery. Trial sequential analysis (TSA) was carried out to assess the reliability of the findings.

## Materials and Methods

This systematic review and meta-analysis was performed in accordance with the PRISMA statement and the recommendations of the Cochrane Collaboration^8,9^ (Supplementary Table S1). The protocol is registered on PROSPERO (registration number: CRD42017059593).

### Search strategy

Two review authors (KP, WRC) independently searched the PubMed, EMBASE, CENTRAL, CNKI, and WanFang databases from inception to March 15, 2017 using MeSH terms combined with text words, without restriction on language. A manual search of the reference lists from relevant articles was also carried out. The search strategies for PubMed, EMBASE, and CENTRAL are summarized in Supplementary Table S2.

### Study selection

Two authors (KP, WRC) independently examined titles and abstracts and reviewed articles to select eligible studies. Inclusion criteria were: (1) study design: randomized controlled trial (RCT); (2) participants: adult patients undergoing arthroscopic knee surgery; (3) comparisons: intra-articular DEX versus saline control, or intra-articular DEX combined with a local anesthetic versus local anesthetic alone; and (4) outcomes: postoperative pain scores, opioid requirement, time to first request for postoperative analgesia, and incidence of postoperative nausea and vomiting (PONV), hypotension, bradycardia, or somnolence. Exclusion criteria were: (1) study design other than a RCT; (2) reviews, letters, abstracts, or editorials; or (3) studies that reported insufficient data. Disagreements about study selection were resolved by group discussion and consensus.

### Data extraction

Two authors (KP, WRC) independently extracted data from each eligible RCTs, including first author, publication year, number of patients, type of anesthesia, type of intraoperative and postoperative analgesia, and outcome measures.

Primary outcome measures were postoperative pain and cumulative opioid consumption. Postoperative pain was measured as pain at rest at postoperative 1, 2, 4, 6, 8, 12, and 24 h and on movement at postoperative 1, 2, and 8 h expressed on a visual analog scale (VAS), where 0 = no pain and 10 = the most severe pain. Opioid consumption during postoperative 0–24 h was expressed in milligrams of morphine equivalent, where intravenous (i.v.) morphine 10 mg = i.v. tramadol 100 mg = i.v. fentanyl 0.1 mg^10,11^. Secondary outcomes were time to first request for postoperative analgesia and incidence of PONV, hypotension, bradycardia, or somnolence.

Disagreements about data extraction were resolved by group discussion and consensus.

### Quality assessment

Two authors (KP, XWM) independently graded the methodological quality of each RCT and the quality of evidence for each outcome measure.

The methodological quality was assessed with the Cochrane risk of bias tool^9^. Seven domains (random sequence generation, allocation concealment, blinding of participants and personnel, blinding of outcome assessment, incomplete outcome data, selective reporting, and other bias [baseline characteristics, funding]) were examined. Risk of bias was categorized as high (one or more domains were high risk), low (all domains were low risk), or unclear (other).

The quality of evidence for each outcome measure was evaluated with Grading of Recommendations Assessment, Development and Evaluation (GRADE) methodology (https://gradepro.org)^12^. Evidence was categorized as high, moderate, low, or very low according to five criteria: risk of bias, inconsistency, indirectness, imprecision, and publication bias.

Publication bias was assessed using Begg’s rank correlation test and Egger’s linear regression test^13,14^.

Disagreements about quality assessment were resolved by group discussion and consensus.

### Statistical analysis

Statistical analyses were performed using STATA 14.0 (Stata Corp, College Station, TX).

To describe the effect size of an intervention, risk ratios (RRs) with 95% confidence intervals (CIs) were calculated for dichotomous data, and weighted mean differences (WMDs) with 95% CIs were calculated for continuous variables. A random-effects model was used in this meta-analysis^15^. Heterogeneity was measured and expressed as *I*^2^, with *I*^2^ > 50% indicating significant heterogeneity^16^. Subgroup analyses were conducted for the outcome measures, stratified by allocation concealment (adequate or unclear), use of local anesthetic (yes or no), type of postoperative analgesic (opioids or none opioids), use of postoperative patient-controlled analgesia (PCA, yes or no), and DEX dose (≤1 µg/kg or >1 µg/kg). Sensitivity analysis was performed by omitting one study at a time to assess the effect of a single comparison on the overall estimates^17^. P < 0.05 denoted statistical significance.

As Type I and Type II errors may arise in a meta-analysis due to small sample size^18^, we assessed the reliability of the current results using TSA (version 0.9.5.5 beta, http://www.ctu.dk/tsa)^18,19^. If the cumulative Z curve crosses the trial sequential monitoring boundary or the futility boundary in the TSA diagram, it is unlikely that an anticipated effect is absent, or that further studies will change the inference. The required information size was calculated using a Type I error of 5%, a power of 80%, and intervention effects in RCTs with adequate allocation concealment^15,20^.

## Results

**Figure 1:**
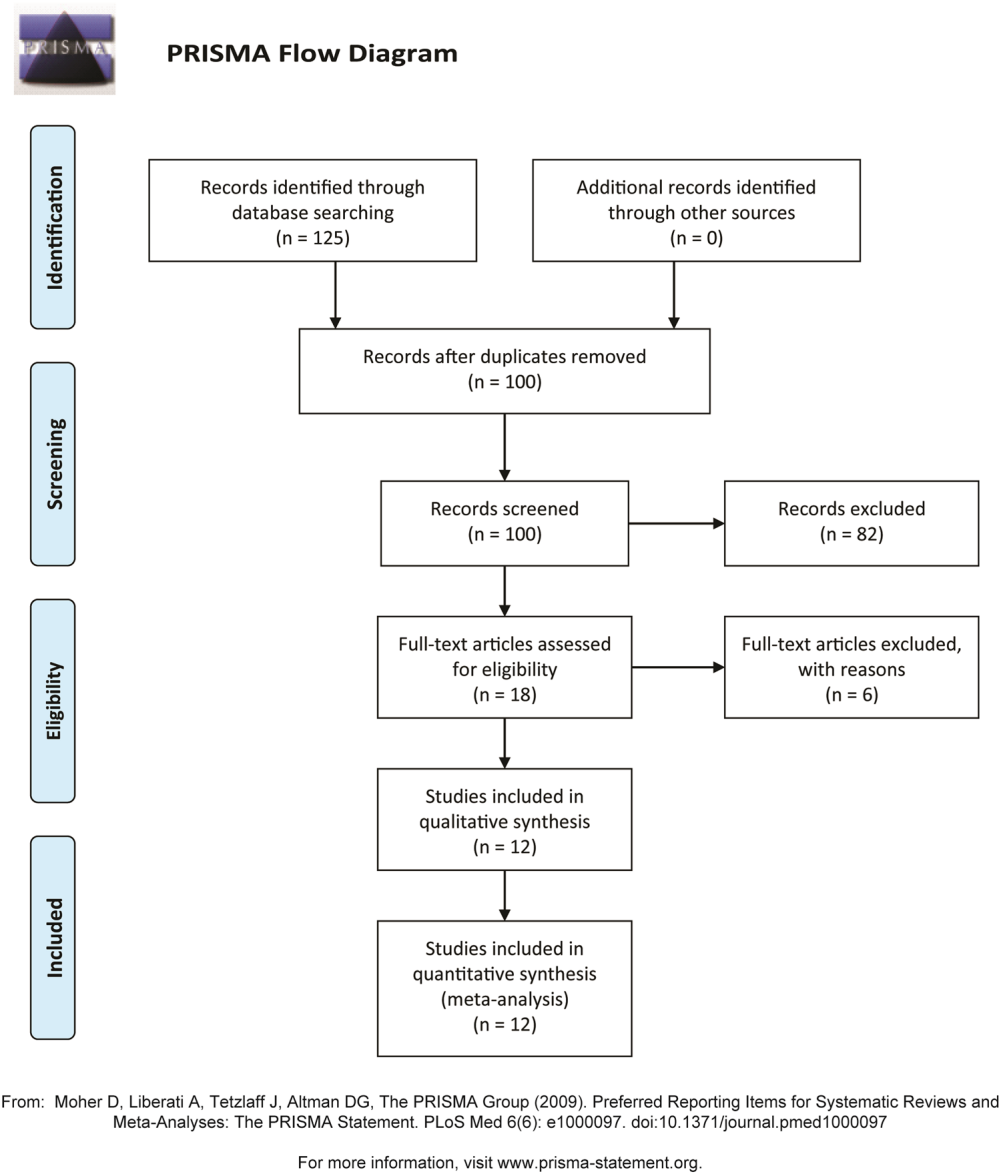
PRISMA flow diagram.

The initial literature search identified 125 articles. Of these, 113 were excluded because they failed to meet the eligibility criteria or were duplicates. A total of 12 RCTs involving 594 participants were finally included in this meta-analysis^7,21–31^. The PRISMA flow diagram is shown in Fig. 1.

**Figure 2:**
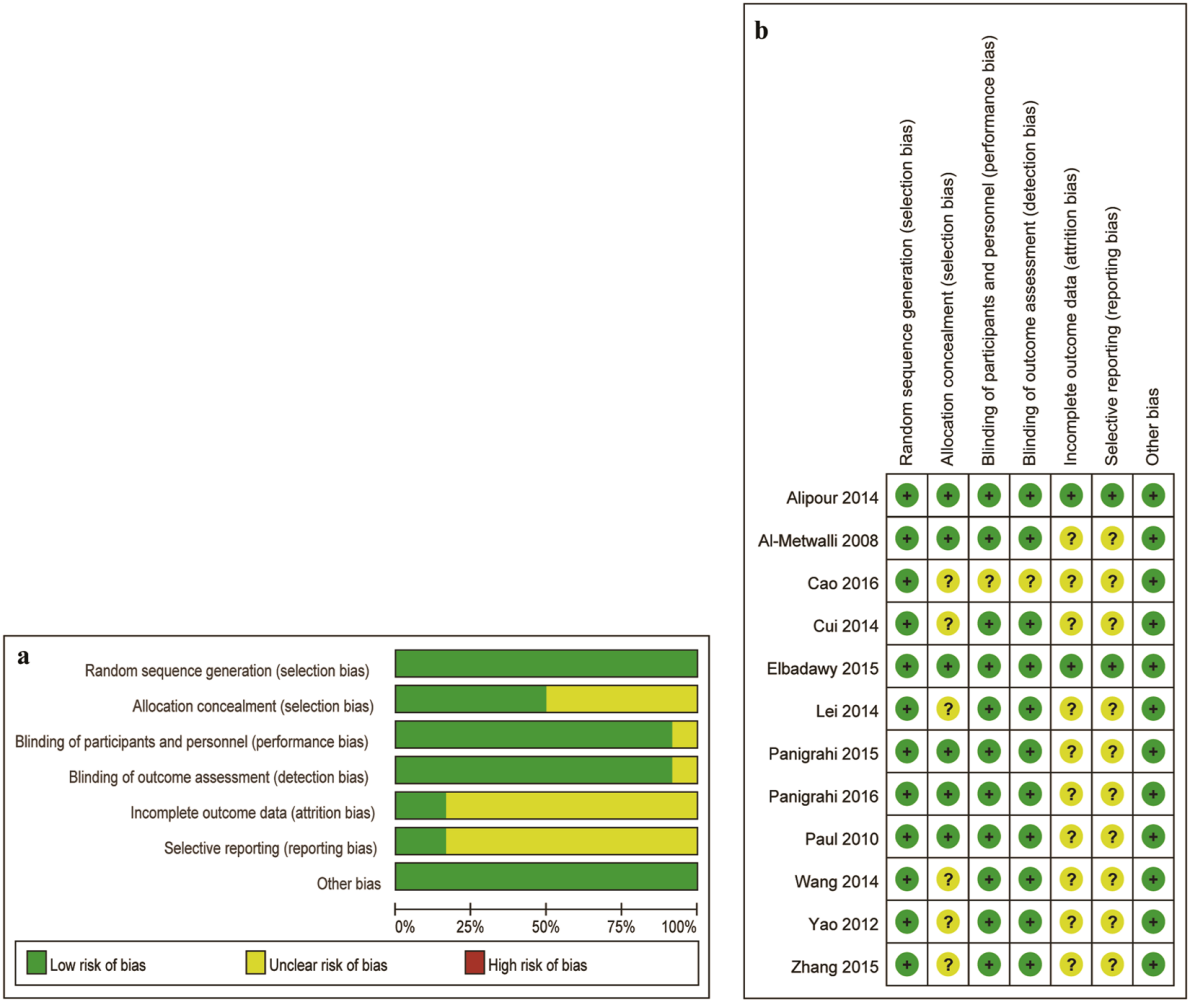
Risk of bias assessment. (**a**) Risk of bias graph; (**b**) risk of bias summary.

The characteristics of the included trials are provided in Table 1. Five trials compared intra-articular DEX with saline^7,21,22,25,30^, and seven trials compared a combination of DEX and a local anesthetic to local anesthetic alone^23,24,26–29,31^. The risk assessment is presented in Fig. 2. All trials were randomized and double-blind, with adequate allocation concealment in 6 trials^7,21,24,26–28^.

**Table 1:** Study characteristics.

**Author, year**	**Country**	**Groups and interventions**	**(n)**	**Age (y), % males**	**Time of treatment**	**Anesthesia**	**Intra/postoperative analgesia**	**Main outcomes**
Al-Metwalli, 2008	Saudi Arabia	1. DEX 1 μg/kg + saline to 20 ml 2. Saline 20 ml	20 20	40.2, 55% 38.2, 65%	Before tourniquet release	General	Fentanyl/diclofenac	Pain VAS at 1–24 h, diclofenac use 0–24 h, time to first analgesia, PONV, hypotension, and bradycardia
Alipour, 2014	Iran	1. DEX 1 µg/kg + saline to 25 ml 2. Saline 25 ml	21 23	30.8, 71.4% 29.4, 73.9%	Before tourniquet release	General	Fentanyl/tramadol	Pain VAS at 1–24 h, tramadol use 0–24 h, time to first analgesia, PONV, hypotension, bradycardia, and pruritus
Cao, 2016	China	1. DEX 100 µg + saline to 15 ml 2. DEX 50 µg + saline to 15 ml 3. Saline 15 ml	20 20 20	42.1, 50% 44.1, 35% 42.2, 40%	Before the end of surgery	General	Fentanyl/flurbiprofen	Pain VAS at 1–24 h, flurbiprofen use 0–24 h, time to first analgesia, PONV, hypotension, bradycardia, and somnolence
Cui, 2014	China	1. DEX 1 µg/kg + 0.25% bupivacaine to 20 ml 2. 0.25% bupivacaine 20 ml	20 20	40.2, 40% 39.4, 4%	Before the end of surgery	General	Fentanyl/PCA with fentanyl	Pain VAS at 1–24 h, fentanyl use 0–24 h, time to first analgesia, PONV, hypotension, bradycardia, and somnolence
Elbadawy, 2015	Egypt	1. DEX 1 µg/kg + 0.25% bupivacaine to 25 ml 2. 0.25% bupivacaine 25 ml	25 25	36.5, 56% 33.6, 64%	Before tourniquet release	General	Fentanyl/paracetamol	Pain VAS at 0.5–12 h, paracetamol use 0–24 h, time to first analgesia, PONV, hypotension, bradycardia, and hallucination
Lei, 2014	China	1. DEX 1 μg/kg + saline to 20 ml 2. Saline 20 ml	20 20	40.0, 40% 39.0, 45%	Before the end of surgery	General	Fentanyl/lornoxicam	Pain VAS at 1–24 h, lornoxicam use 0–24 h, time to first analgesia, PONV, hypotension, and bradycardia
Panigrahi, 2015	India	1. DEX 1 µg/kg + 0.2% ropivacaine to 20 ml 2. DEX 2 µg/kg + 0.2% ropivacaine to 20 ml 3. 0.2% ropivacaine 20 ml	20 20 20	31.7, 75% 32.3, 65% 30.8, 70%	Before tourniquet release	Spinal	Bupivacaine/diclofenac	Pain VAS at 1–24 h, diclofenac use 0–24 h, time to first analgesia, PONV, hypotension, and bradycardia
Panigrahi, 2016	India	1. DEX 1 µg/kg + 0.2% ropivacaine to 20 ml 2. 0.2% ropivacaine 20 ml	20 20	31.7, 75% 30.8, 70%	Before tourniquet release	Spinal	Bupivacaine/diclofenac	Pain VAS at 1–24 h, diclofenac use 0–24 h, time to first analgesia, PONV, hypotension, and bradycardia
Paul, 2010	India	1. DEX 100 µg + 0.25% ropivacaine to 20 ml 2. 0.25% ropivacaine 20 ml	30 30	41.4, 70% 39.8, 73%	Before arthroscope removal	General	Fentanyl/PCA with fentanyl	Pain VAS at 1–18 h, fentanyl use 0–24 h, time to first analgesia, PONV, hypotension, bradycardia, and somnolence
Wang, 2014	China	1. DEX 1 µg/kg + 0.25% ropivacaine to 20 ml 2. 0.25% ropivacaine 20 ml	30 30	40.0, 46% 40.0, 56%	Before tourniquet release	General	Fentanyl/fentanyl	Pain VAS at 1–24 h, fentanyl use 0–24 h, time to first analgesia, PONV, hypotension, bradycardia, and hypoxemia
Yao, 2012	China	1. DEX 0.7 μg/kg + saline to 15 ml 2. Saline 15 ml	20 20	40.2, 55% 39.2, 65%	Before tourniquet release	General	Fentanyl/tramadol	Pain VAS at 1–12 h, tramadol use 0–24 h, time to first analgesia, PONV, hypotension, and bradycardia
Zhang, 2015	China	1. DEX 100 µg + 0.25% ropivacaine to 20 ml 2. 0.25% ropivacaine 20 ml	30 30	40.4, 63% 38.9, 66%	Before the end of surgery	General	Fentanyl/PCA with fentanyl	Pain VAS at 1–24 h, fentanyl use 0–24 h, time to first analgesia, PONV, hypotension, bradycardia, and somnolence

DEX, dexmedetomidine; VAS, visual analogue scale score; PONV, postoperative nausea and vomiting; PCA, patient controlled analgesia.

**Figure 3:**
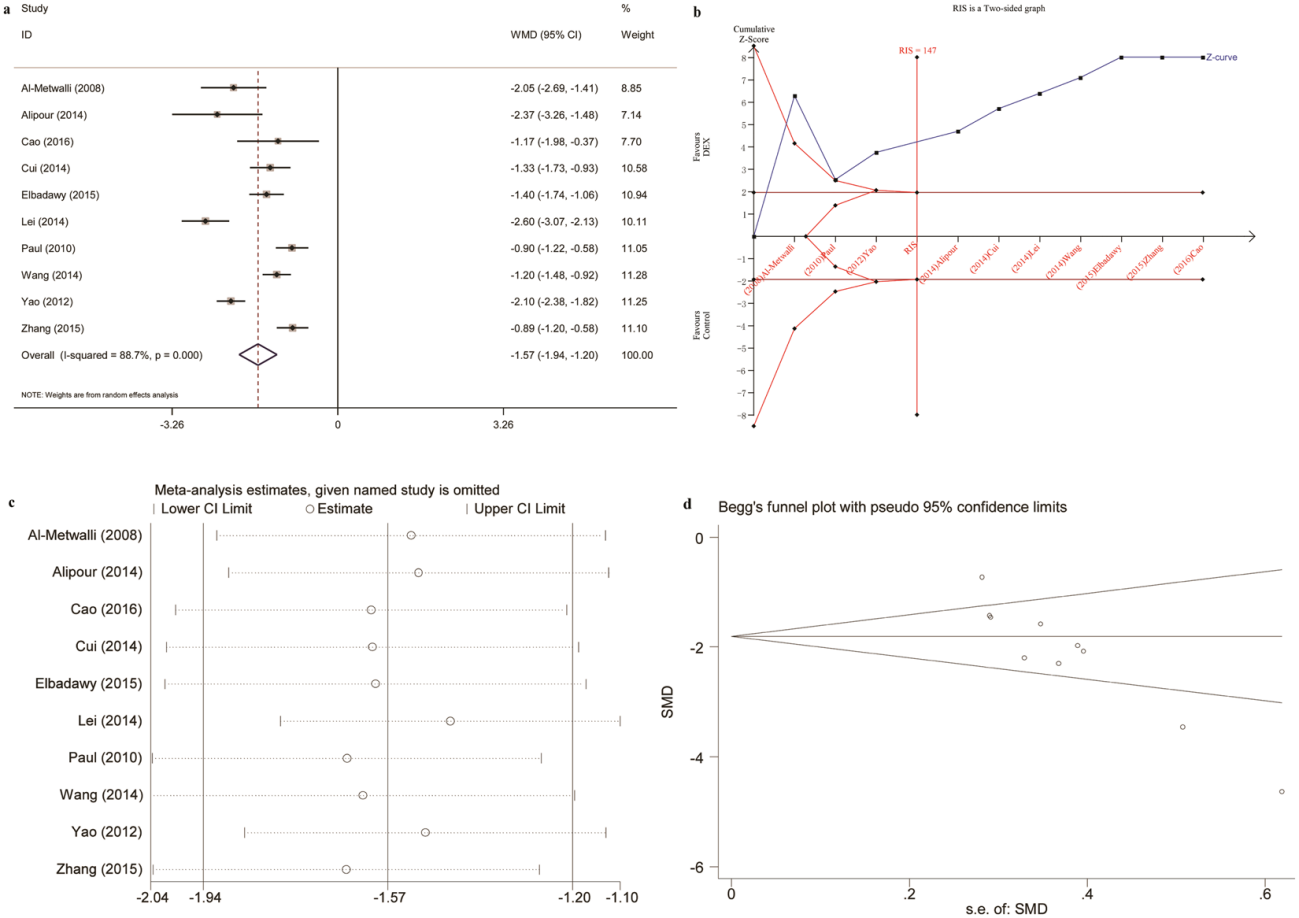
Intra-articular DEX versus control in knee arthroscopy: pain intensity at rest at 1 h postoperatively. (**a**) Forest plot; (**b**) Trial sequential analysis; (**c**) Sensitivity analysis; (**d**) Begg’s funnel plot. DEX, dexmedetomidine; WMD, weighted mean difference; RIS, required information size.

The primary and secondary outcomes are summarized in Table 2. The meta-analysis demonstrated significantly lower postoperative pain intensity at rest (1 h postoperatively: WMD, −1.57 [95% CI −1.94 to −1.20]; P = 0.0001) in patients treated with intra-articular DEX or a combination of DEX and local anesthetic compared to saline or local anesthetic alone (Fig. 3a). This statistically significant difference was also observed at postoperative 2 to 24 h and at 3 time points for pain intensity on movement. According to the TSA, the required information size of 147 patients was reached and the cumulative Z curve crossed the trial sequential monitoring boundary, indicating there is sufficient evidence for this outcome (Fig. 3b). Sensitivity analysis demonstrated these findings were robust (Fig. 3c), with pooled WMDs ranging from −1.65 (95% CI −2.04 to −1.26) to −1.44 (95% CI −1.78 to −1.10). Begg’s funnel plot (P = 0.002, Fig. 3d) and Egger’s test (P < 0.0001) indicated publication bias.

**Table 2:** Outcomes.

Outcomes	References	DEX (n)	Control (n)	Estimated benefit [95% CI]	*P* value	*I*^2^ (%)
***Pain intensity at rest***						
1 h postoperatively	^7,21–25,28–31^	256	238	WMD = −1.57 [−1.94, −1.20]	0.00001	89
2 h postoperatively	^7,22–25,28–31^	235	215	WMD = −1.46 [−1.82, −1.10]	0.00001	89
4 h postoperatively	^7,24,25,29–31^	145	145	WMD = −1.37 [−1.76, −0.97]	0.00001	84
6 h postoperatively	^7,21,23–25,28,30,31^	186	188	WMD = −1.26 [−1.61, −0.92]	0.00001	83
8 h postoperatively	^7,22,29–31^	140	120	WMD = −1.02 [−1.41, −0.64]	0.00001	70
12 h postoperatively	^7,21,24,25,29–31^	166	168	WMD = −0.55 [−0.88, −0.23]	0.0009	69
24 h postoperatively	^7,21–23,25,29,31^	181	163	WMD = −0.34 [−0.68, 0.00]	0.05	59
***Pain intensity on movement***						
1 h postoperatively	^22,29,30^	90	70	WMD = −1.71 [−2.39, −1.03]	0.00001	86
2 h postoperatively	^22,29,30^	90	70	WMD = −1.80 [−2.25, −1.36]	0.00001	70
8 h postoperatively	^22,29,30^	90	70	WMD = −1.29 [−1.57, −1.02]	0.00001	32
***Other outcomes***						
Morphine-equivalents 0–24 h (mg)	^21,23,28–31^	151	153	WMD = −8.54 [−11.96, −5.13]	0.00001	95
Time to first analgesic request (min)	^7,21–31^	316	278	WMD = 257.57 [209.86, 305.28]	0.00001	93
PONV	^7,21–31^	316	278	RR = 1.37 [0.52, 3.62]	0.52	0
Hypotension	^7,21–31^	316	278	RR = 2.20 [0.67, 7.23]	0.19	0
Bradycardia	^7,21–31^	316	278	RR = 2.20 [0.81, 5.97]	0.12	0
Somnolence	^22,23,28,31^	120	100	RR = 1.54 [0.07, 36.11]	0.79	0

DEX group vs. control group for all comparisons.

Pain intensity was scored with a visual analog scale as: 0 = no pain, 10 = the most severe pain imaginable.

Morphine-equivalents were calculated as: morphine 10 mg = tramadol 100 mg = fentanyl 0.1 mg, intravenously.

DEX, dexmedetomidine; PONV, postoperative nausea and vomiting; WMD, weighted mean difference; RR, risk ratio; CI, confidence interval.

**Figure 4:**
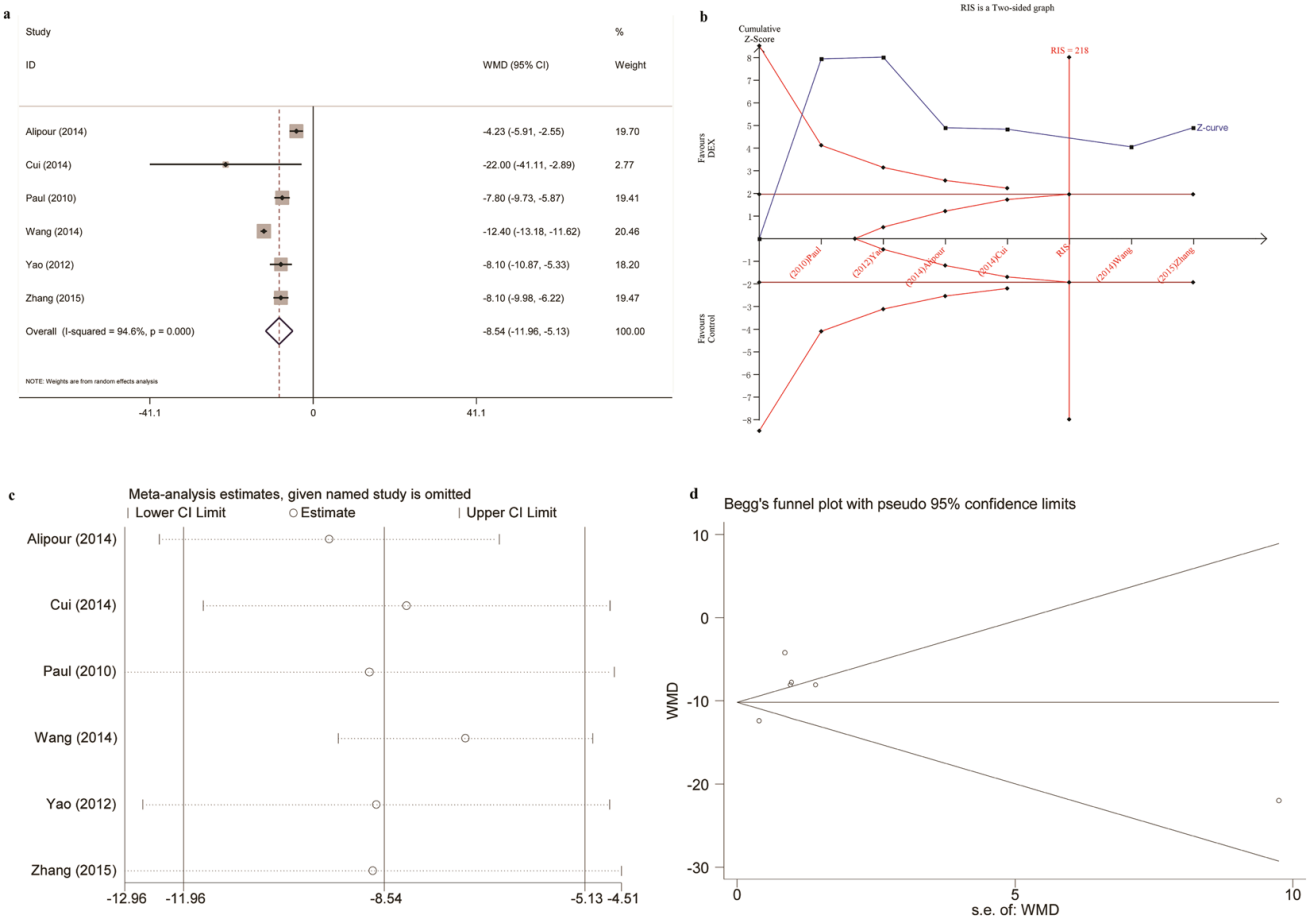
Intra-articular DEX versus control in knee arthroscopy: morphine-equivalents 0–24 h postoperatively. (**a**) Forest plot; (**b**) Trial sequential analysis; (**c**) Sensitivity analysis; (**d**) Begg’s funnel plot. DEX, dexmedetomidine; WMD, weighted mean difference; RIS, required information size.

At postoperative 0–24 h, opioid consumption was significantly reduced with a WMD of −8.54 mg morphine-equivalents in patients treated with intra-articular DEX or a combination of DEX and local anesthetic compared to saline or local anesthetic alone (Fig. 4a,b). Sensitivity analysis demonstrated these findings were robust, with pooled WMDs ranging from −9.48 mg (95% CI −12.37 to −6.58) to −7.16 mg (95% CI −9.33 to −5.00) (Fig. 4c). No evidence of publication bias was observed on Begg’s funnel plot (P = 0.707, Fig. 4d) or Egger’s test (P = 0.328).

**Figure 5:**
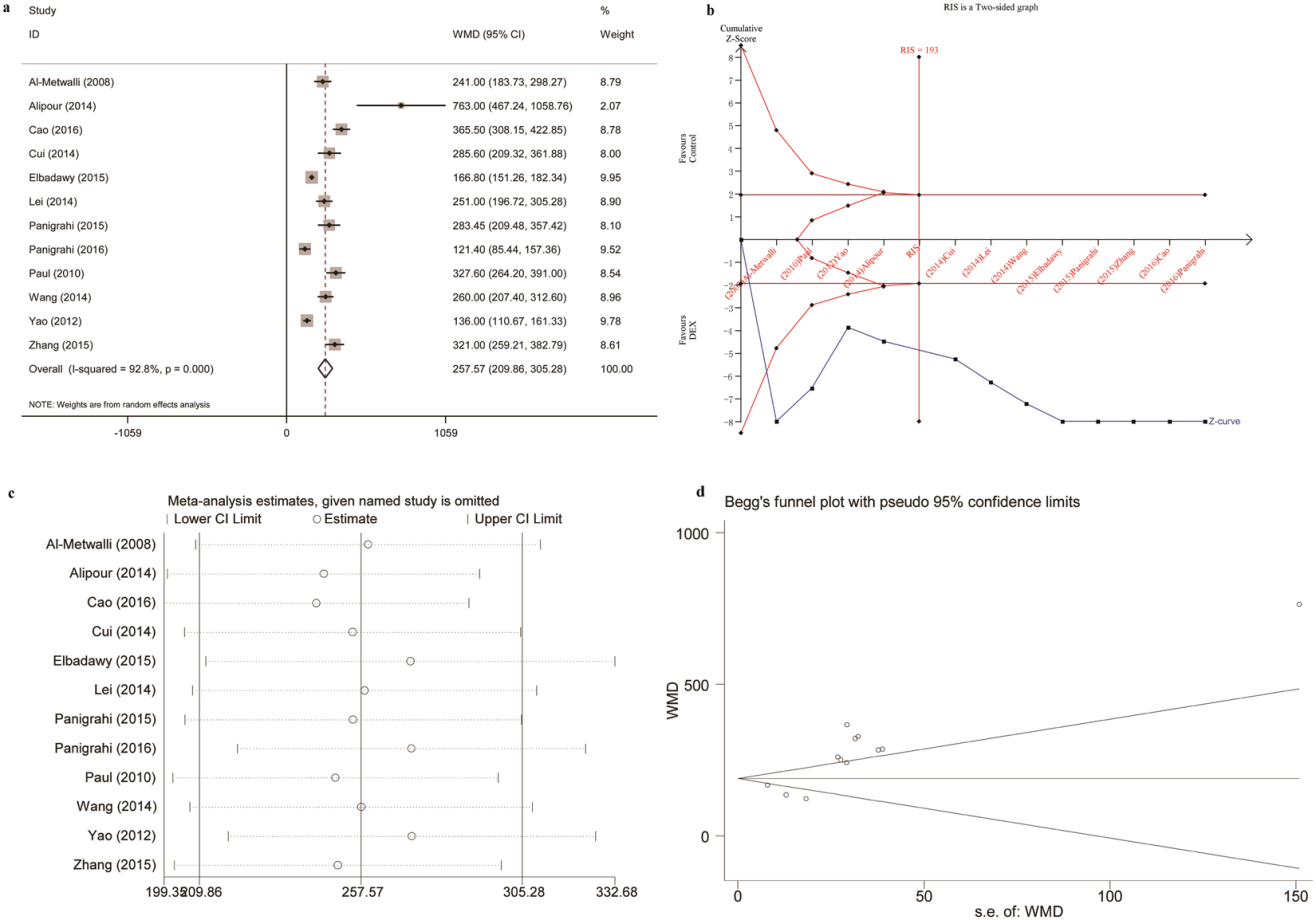
Intra-articular DEX versus control in knee arthroscopy: time to first analgesic request. (**a**) Forest plot; (**b**) Trial sequential analysis; (**c**) Sensitivity analysis; (**d**) Begg’s funnel plot. DEX, dexmedetomidine; WMD, weighted mean difference; RIS, required information size.

The use of intra-articular DEX or a combination of DEX and local anesthetic led to a longer time to first request for postoperative analgesia compared to saline or local anesthetic alone (WMD = 257.57 min) (Fig. 5a,b). Sensitivity analysis demonstrated these findings were robust, with pooled WMDs ranging from 244.42 min (95% CI 199.35 to 289.49) to 272.65 min (95% CI 218.34 to 326.96) (Fig. 5c). Begg’s funnel plot (P = 0.064, Fig. 5d) and Egger’s test (P = 0.002) indicated publication bias.

**Figure 6:**
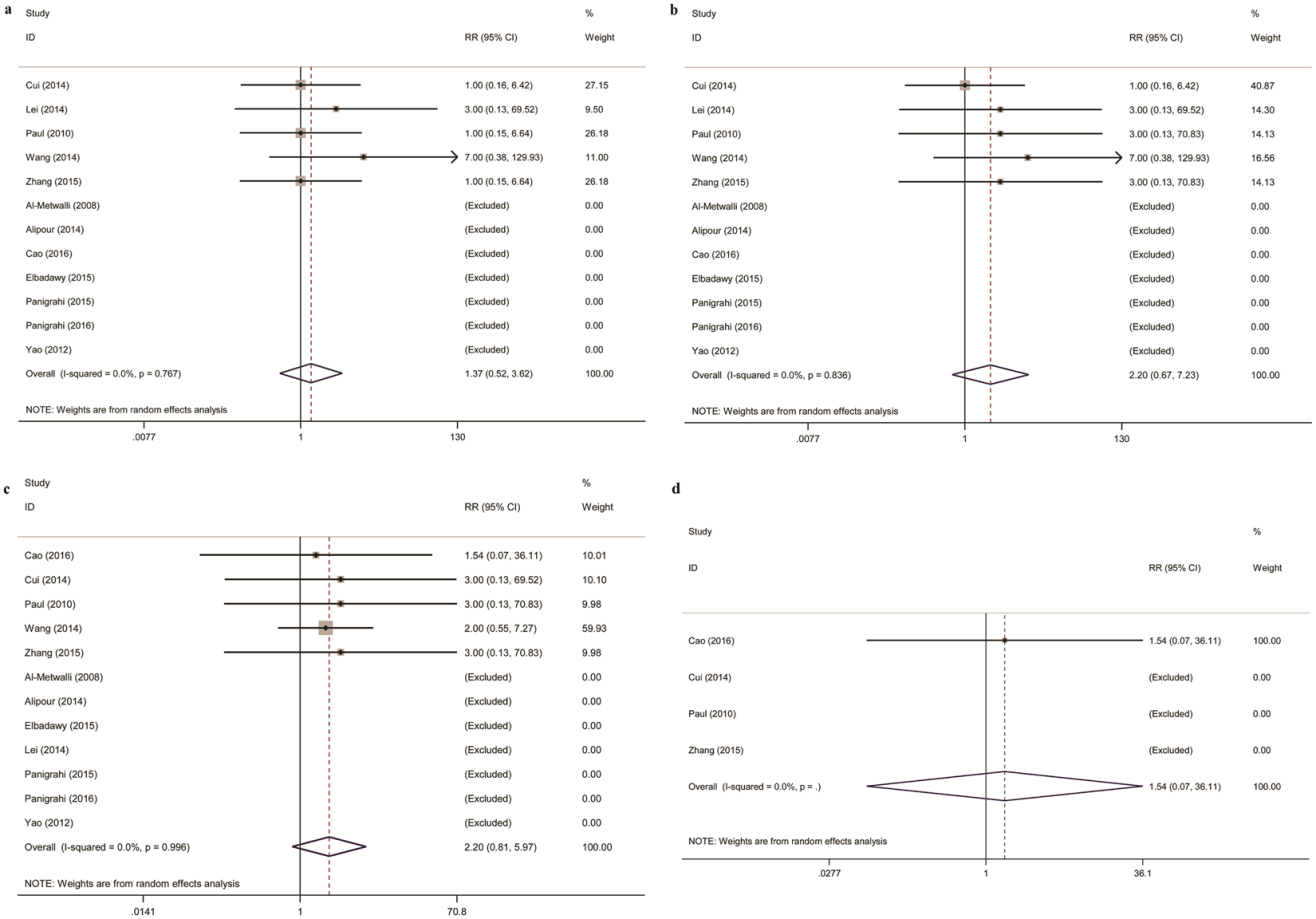
Intra-articular DEX versus control in knee arthroscopy: adverse effects. (**a**) Postoperative nausea and vomiting; (**b**) Hypotension; (**c**) Bradycardia; (**d**) Somnolence. DEX, dexmedetomidine; WMD, weighted mean difference.

For adverse effects, there were no significant differences in the incidence of PONV, hypotension, bradycardia, or somnolence between the DEX and control groups (Fig. 6a–d).

Subgroup analyses are shown in Table 3. Use of local anesthetic (yes or no) and postoperative PCA (yes or no) and DEX dose (≤1 µg/kg or >1 µg/kg) may account for the heterogeneity in some of the findings.

**Table 3:** Subgroup analyses.

**Subgroups**	**No. of studies**	**Mean difference [95% CI]**	***P*** **value**	***I*** ^**2**^ **(%)**	**Between subgroup significance**
***Pain intensity at rest at 1*** ***h postoperatively***					
Total	10	−1.57 [−1.94, −1.20]	0.00001	89	
Allocation concealment					
Adequate	4	−1.58 [−2.15, −1.01]	0.00001	83	0.95
Unclear	6	−1.55 [−2.07, −1.04]	0.00001	92	
Local anesthetic use					
Yes	5	−1.13 [−1.34, −0.93]	0.00001	50	0.0001
No	5	−2.11 [−2.50, −1.73]	0.00001	59	
Postoperative analgesics					
Opioids	6	−1.41 [−1.86, −0.96]	0.00001	90	0.31
None opioids	4	−1.83 [−2.50, −1.15]	0.00001	85	
Postoperative PCA use					
Yes	3	−1.01 [−1.27, −0.76]	0.00001	43	0.002
No	7	−1.83 [−2.26, −1.39]	0.00001	86	
DEX dosage					
≤1 µg/kg	7	−1.82 [−2.23, −1.40]	0.00001	86	0.0001
>1 µg/kg	3	−0.91 [−1.13, −0.70]	0.00001	0	
***Morphine-equivalents 0–*** **2** ***4 h postoperatively (mg)***					
Total	6	−8.54 [−11.96, −5.13]	0.00001	95	
Allocation concealment					
Adequate	2	−5.98 [−9.48, −2.48]	0.0008	87	0.10
Unclear	4	−10.02 [−13.37, −6.68]	0.00001	88	
Local anesthetics use					
Yes	4	−9.88 [−13.27, −6.49]	0.00001	91	0.13
No	2	−6.00 [−9.78, −2.22]	0.002	82	
Postoperative PCA use					
Yes	3	−8.03 [−9.44, −6.62]	0.00001	5	0.94
No	3	−8.28 [−14.06, −2.50]	0.005	97	
DEX dosage					
≤1 µg/kg	4	−9.20 [−14.78, −3.62]	0.001	96	0.67
>1 µg/kg	2	−7.95 [−9.30, −6.61]	0.00001	0	
***Time to first analgesic request (min)***					
Total	12	257.57 [209.86, 305.28]	0.00001	93	
Allocation concealment					
Adequate	6	246.70 [177.68, 315.72]	0.00001	92	0.70
Unclear	6	268.03 [185.08, 350.97]	0.00001	94	
Local anesthetics use					
Yes	7	247.75 [187.67, 307.83]	0.00001	92	0.49
No	5	291.85 [182.51, 401.20]	0.00001	95	
Postoperative analgesics					
Opioids	6	298.74 [202.20, 395.27]	0.00001	94	0.28
None opioids	6	234.38 [170.64, 298.12]	0.00001	93	
Postoperative PCA use					
Yes	3	314.49 [276.22, 352.77]	0.00001	0	0.02
No	9	236.52 [186.24, 286.80]	0.00001	93	
DEX dosage					
≤1 µg/kg	8	213.20 [169.44, 256.96]	0.00001	89	0.0001
>1 µg/kg	4	329.21 [297.12, 361.30]	0.00001	3	
Anesthesia					
General	10	271.55 [217.40, 325.71]	0.00001	93	0.40
Spinal	2	199.07 [40.40, 357.74]	0.01	93	

DEX, dexmedetomidine; PCA, patient-controlled analgesia; CI: confidence interval.

The GRADE evidence profiles for the outcome measures are shown in Table 4. The level of evidence was moderate for pain intensity at rest at postoperative 1 h, and low for pain intensity on movement at postoperative 1 h, morphine-equivalents at postoperative 0–24 h, time to first request for postoperative analgesia, PONV, hypotension, and bradycardia.

**Table 4:** GRADE evidence profile.

**Quality assessment**							**№ of patients**		**Effect**		**Quality**	**Importance**
**№ of studies**	**Study design**	**Risk of bias**	**Inconsistency**	**Indirectness**	**Imprecision**	**Other considerations**	**DEX**	**Control**	**Relative (95% CI)**	**Absolute (95% CI)**		
Pain intensity at rest at 1 h postoperatively												
10	randomised trials	not serious	serious^a^	not serious	not serious	none	256	238	—	MD **1.57 lower** (1.94 lower to 1.2 lower)	⨁⨁⨁◯ MODERATE	IMPORTANT
Pain intensity on movement at 1 h postoperatively												
3	randomised trials	serious^b^	serious^c^	not serious	not serious	none	90	70	—	MD **1.71 lower** (2.39 lower to 1.03 lower)	⨁⨁◯◯ LOW	IMPORTANT
Morphine-equivalents 0–24 h (mg)												
6	randomised trials	not serious	very serious^d^	not serious	not serious	none	151	153	—	MD **8.54 lower** (11.96 lower to 5.13 lower)	⨁⨁◯◯ LOW	IMPORTANT
Time to first analgesic request (min)												
12	randomised trials	not serious	very serious^e^	not serious	not serious	none	316	278	—	MD **257.57 higher** (209.86 higher to 305.28 higher)	⨁⨁◯◯ LOW	IMPORTANT
Postoperative nausea and vomiting												
12	randomised trials	not serious	not serious	not serious	very serious ^f^	none	10/316 (3.2%)	6/278 (2.2%)	RR 1.37 (0.52 to 3.62)	**8 more per 1,000** (from 10 fewer to 57 more)	⨁⨁◯◯ LOW	IMPORTANT
Hypotension												
12	randomised trials	not serious	not serious	not serious	very serious ^g^	none	8/316 (2.5%)	2/278 (0.7%)	RR 2.20 (0.67 to 7.23)	**9 more per 1,000** (from 2 fewer to 45 more)	⨁⨁◯◯ LOW	IMPORTANT
Bradycardia												
12	randomised trials	not serious	not serious	not serious	very serious ^h^	none	10/316 (3.2%)	3/278 (1.1%)	RR 2.20 (0.81 to 5.97)	**13 more per 1,000** (from 2 fewer to 54 more)	⨁⨁◯◯ LOW	IMPORTANT

DEX, dexmedetomidine; CI, confidence interval; MD, mean difference; RR, risk ratio.

^a^Heterogeneity: *I*^2^ = 89%.

^b^All the trials were judged to be at unclear risk of bias.

^c^Heterogeneity: *I*^2^ = 86%.

^d^Heterogeneity: *I*^*2*^ = 95%.

^e^Heterogeneity: *I*^*2*^ = 93%.

^f^RR with 95% CI: 3.00 (0.13–69.52) for one trial and 7.00 (0.38–129.93) for another.

^g^RR with 95% CI: 3.00 (0.13–69.52), 3.00 (0.13–70.83), and 7.00 (0.38–129.93) for three trials.

^h^RR with 95% CI: 1.54 (0.07–36.11), 3.00 (0.13–69.52), and 3.00 (0.13–70.83) for three trials.

## Discussion

In this meta-analysis, intra-articular DEX use significantly decreased postoperative pain and opioid consumption in patients undergoing arthroscopic knee surgery. The analgesic effect of DEX was also indicated by a longer time to first request for postoperative analgesia. The evidence for each outcome measure was verified by TSA. No difference was found in the incidence of PONV, hypotension, or bradycardia between the DEX and control groups.

Arthroscopic surgery is a minimally invasive orthopedic surgical procedure. However, postoperative pain can be underestimated. Al-Metwalli *et al*. showed that patients treated with intra-articular and i.v. saline reported pain intensity of 5 points on a VAS up to 12 h postoperatively^7^. This pain, if left untreated, delays patient discharge and postoperative rehabilitation. Considering the adverse effects associated with systemic opioid use, intra-articular analgesia administration is simple and may provide a better alternative. In this meta-analysis, DEX treatment decreased VAS pain scores by 1.57 points at rest and 1.71 points on movement at postoperative 1 h. The reduction in postoperative pain decreased to 0.34 points at 24 h, but the difference remained significant. Further, morphine-equivalents were 8.54 mg lower and the time to first request for postoperative analgesia was 257.57 min longer in the DEX treatment group compared to the control group. To the authors’ knowledge, these are the first data to demonstrate that the analgesic effects of intra-articular DEX occur mainly in the early postoperative period.

The mechanism underlying the effects of intra-articular DEX is unknown. There may be direct local action, but central action by systemic absorption should not be excluded. A recent study revealed simultaneous perineural administration of clonidine and DEX prolonged sensory and motor blockade by local anesthetics^32,33^. Supraspinal, spinal, and peripheral mechanisms could be involved in the analgesic effects of α2 adrenergic receptor agonists^34^. Similar to clonidine, DEX acts on presynaptic receptors and inhibits the release of norepinephrine at peripheral afferent nociceptors^6^. Some evidence suggests that α2 adrenoceptor stimulation causes analgesia by facilitating inhibitory synaptic responses in the superficial dorsal horn^35^. Notably, the effects of DEX are not limited to its interactions with α2 adrenergic receptors. A direct inhibition of tetrodotoxin-resistant Na^+^ channels may contribute to the antinociceptive effects of clonidine and DEX when used in addition to local anesthesia^36^. Another study indicated that DEX inhibited neuronal delayed-rectifier potassium currents and sodium currents to produce local anesthetic effects^37^.

Systemic DEX infusion potentiates intra- and postoperative analgesia, and perineural DEX may improve the onset and quality of neuraxial and peripheral nerve block^33,38^. However, hemodynamic changes such as hypotension, bradycardia, or both have been reported. In this meta-analysis, intra-articular DEX did not increase the incidence of hypotension or bradycardia compared to that in the control group. The low incidence of adverse effects may be related to the lack of vessels in the articular surface and the relatively small dose of DEX administered.

This meta-analysis has several limitations. First, only 12 trials met the inclusion criteria, and the number of patients was relatively small. Second, substantial heterogeneity was found for some outcome measures. According to subgroup analyses, inconsistencies in the use of local anesthetic and in the postoperative PCA and DEX doses may account for the heterogeneity. Third, although TSA indicated sufficient evidence for the conclusions, the level of evidence achieved by the GRADE methodology was low or moderate. Therefore, the current results should be interpreted with caution. Finally, there are no data on the long-term effects of intra-articular DEX administration; therefore, this study is only relevant to the early postoperative period, specifically, up to postoperative 24 h. Consequently, further well-conducted prospective studies with adequate power to evaluate short-and long-term outcomes after intra-articular administration of DEX are warranted.

In conclusion, intra-articular administration of DEX improved pain outcomes in the early postoperative period after knee arthroscopy. More evidence is required to confirm these findings.

## Electronic supplementary material

Supplementary Information
